# Hidden in Muscles: A Case Study of Intramuscular Lipoma

**DOI:** 10.7759/cureus.61278

**Published:** 2024-05-28

**Authors:** Ashna Nagpal, Pankaj Gharde, Pratik S Navandhar, Chahat Singh, Bhagyesh Sapkale

**Affiliations:** 1 General Surgery, Jawaharlal Nehru Medical College, Datta Meghe Institute of Higher Education and Research, Wardha, IND; 2 Medicine, Jawaharlal Nehru Medical College, Datta Meghe Institute of Higher Education and Research, Wardha, IND

**Keywords:** anatomical location, recurrence risk, surgical approach, soft tissue mass, histopathological examination, mri imaging, lipoma excision, surgical intervention, case study, intramuscular lipoma

## Abstract

This case report presents a 64-year-old male with a giant intramuscular lipoma on the right lumbar region's latissimus dorsi muscle. The patient presented with painless swelling, which gradually increased over six years. Magnetic resonance imaging (MRI) confirmed the presence of the lipoma, prompting surgical intervention. The surgical procedure involved meticulous dissection and complete excision of the tumor. Histopathological examination validated the diagnosis. Comparative analyses with similar cases highlighted variations in surgical approaches and the challenges in managing intramuscular lipomas. This case emphasizes the importance of considering intramuscular lipomas in soft tissue mass differentials and the significance of comprehensive management strategies for optimal patient outcomes.

## Introduction

A lipoma is a fatty tissue growth beneath the skin [1]. Lipomas feel rubbery, not firm, and move quickly when touched [1]. There are several different types of lipomas, such as fibrolipoma (fat with fibrous tissue), angiolipoma (fat plus numerous blood vessels), hibernoma (brown fat, more frequently observed in youngsters), and conventional lipoma (common, mature white fat) [2]. A highly uncommon type of lipoma called intramuscular lipoma is found deep within the muscle fibers and exhibits symptoms of invading the nearby muscles [3]. Among the upper body's muscles, the latissimus dorsi is the largest, and it runs from the lower spine to the upper arm [4]. A benign tumor made of fatty tissue that grows within the latissimus dorsi muscle is known as a lipoma of the muscle [5]. Lipoma excision is a procedure of surgery to remove a lipoma [1].

## Case presentation

**Figure 1:**
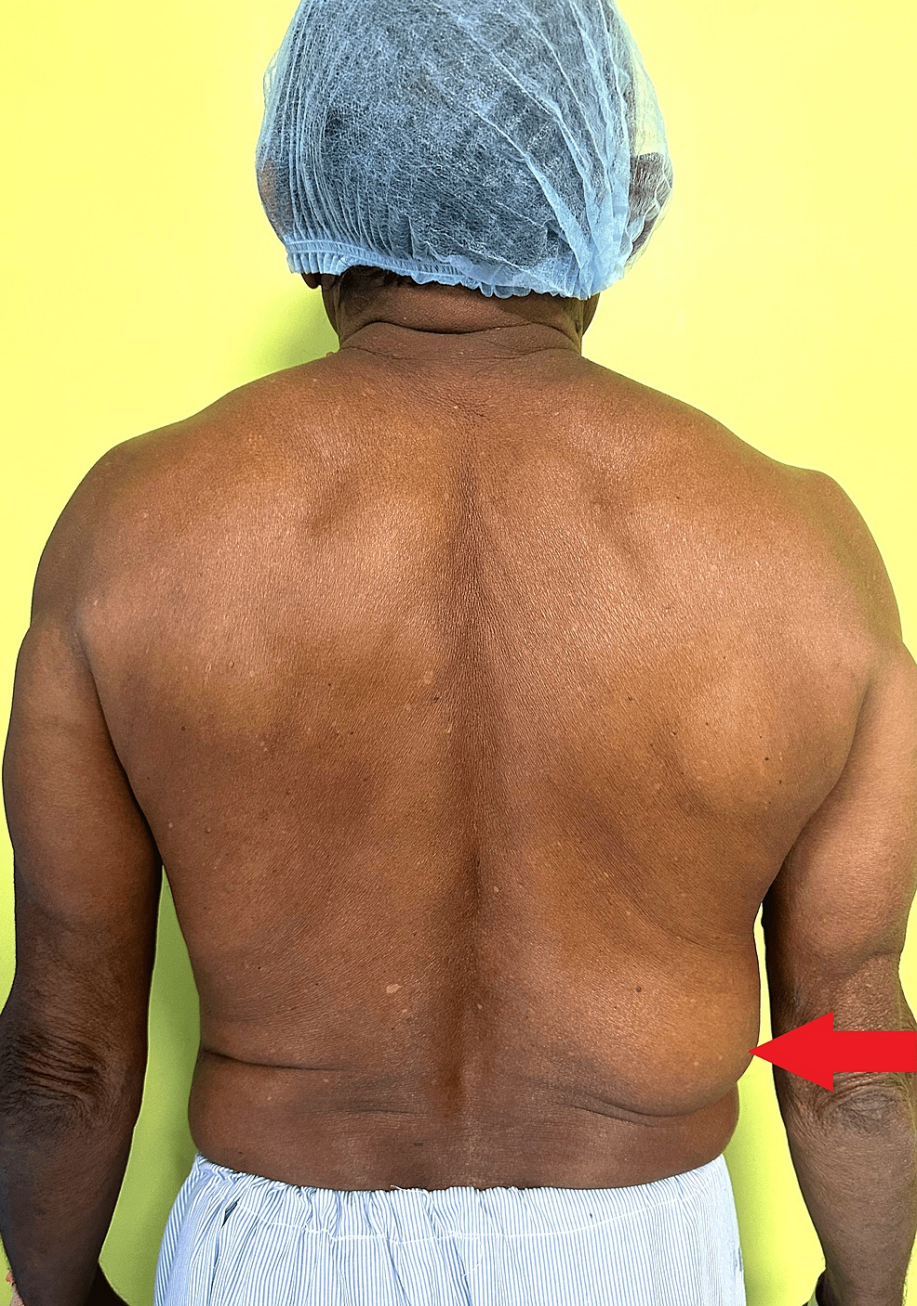
Swelling in the right lumbar region Swelling in the right lumbar region is pointed with the red-colored arrow

A 64-year-old male patient arrived at Acharya Vinoba Bhave Rural Hospital (AVBRH) complaining of swelling in his right lumbar region, as shown in Figure 1, that had persisted without pain for the past six years. There is no family history of lipoma, and no other locations of lipoma are noted in the given case. The swelling was initially noticed while taking a bath; initially small at 2 cm, the swelling had gradually expanded to a significant 10 x 8 x 8 cm mass.

**Figure 2:**
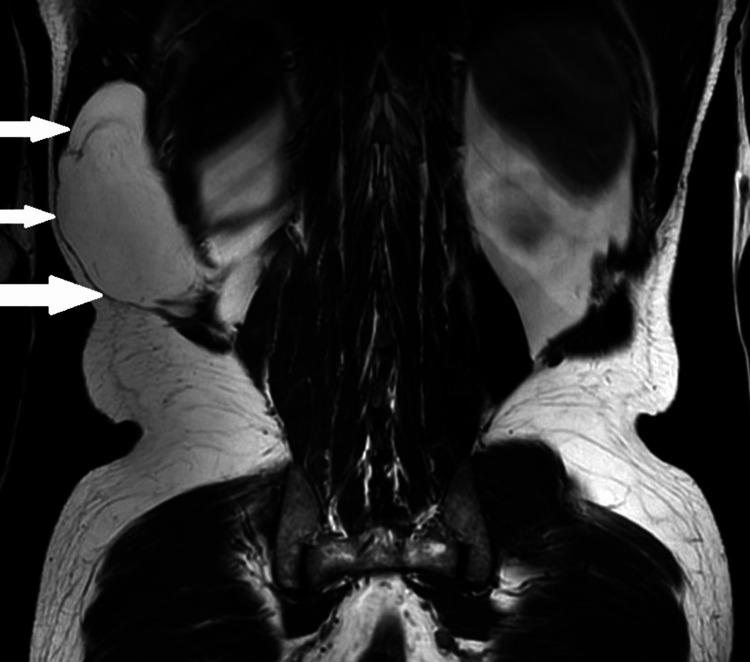
MRI showing intramuscular lipoma MRI: Magnetic resonance imaging MRI shows an encapsulated mass within the right latissimus dorsi muscle, with white arrows pointing toward the intramuscular lipoma

Physical examination revealed a palpable, nontender, mobile mass palpated in the subcutaneous tissue without signs of inflammation or skin changes overlying the swelling. Magnetic resonance imaging (MRI) findings confirmed the presence of an encapsulated mass within the right latissimus dorsi muscle, consistent with an intramuscular lipoma, as shown in Figure 2. There were no restrictions to the range of movements of latissimus dorsi due to this tumor.

Following the confirmation of an intramuscular lipoma through MRI imaging, surgical intervention was deemed necessary to address the swelling in the right lumbar region. The procedure commenced with the patient placed in a left lateral position under general anesthesia. A standard aseptic technique was employed, and the surgical field was prepared and draped accordingly. The first step involved making an appropriate horizontal incision over the site of the swelling in the right lumbar region. Careful consideration was given to ensure optimal exposure while minimizing damage to surrounding tissues. Once the incision was made, sharp dissection was initiated to dissect through the layers of subcutaneous tissue until reaching the fascia surrounding the right latissimus dorsi muscle. The encapsulated mass within the right latissimus dorsi muscle was visualized with meticulous dissection. During the dissection process, special care was taken to identify and preserve important structures, such as nerves and blood vessels. Once fully exposed, the tumor was carefully dissected free from its surrounding tissue planes. The next step involved excising the intramuscular lipoma in its entirety. This was achieved by dissecting around the tumor margin and ensuring complete removal while avoiding any spillage of its contents.

Hemostasis was meticulously maintained throughout the procedure to minimize bleeding and optimize visualization. After the tumor was completely excised, the wound was thoroughly irrigated with a sterile saline solution to remove debris or residual tissue. Closure of the wound was performed in layers using appropriate sutures to approximate the deep fascial layer, followed by subcutaneous tissue and skin closure with absorbable sutures as deemed appropriate. Following closure, a sterile dressing was applied to the surgical site, and the patient was transferred to the recovery area for postoperative monitoring. Pain management and wound care instructions were provided, and the patient was scheduled for follow-up appointments to assess healing progress and monitor for any signs of recurrence or complications. In summary, the surgical intervention for the intramuscular lipoma involved meticulous dissection, complete tumor excision, and careful wound closure to ensure optimal outcomes for the patient.

**Figure 3:**
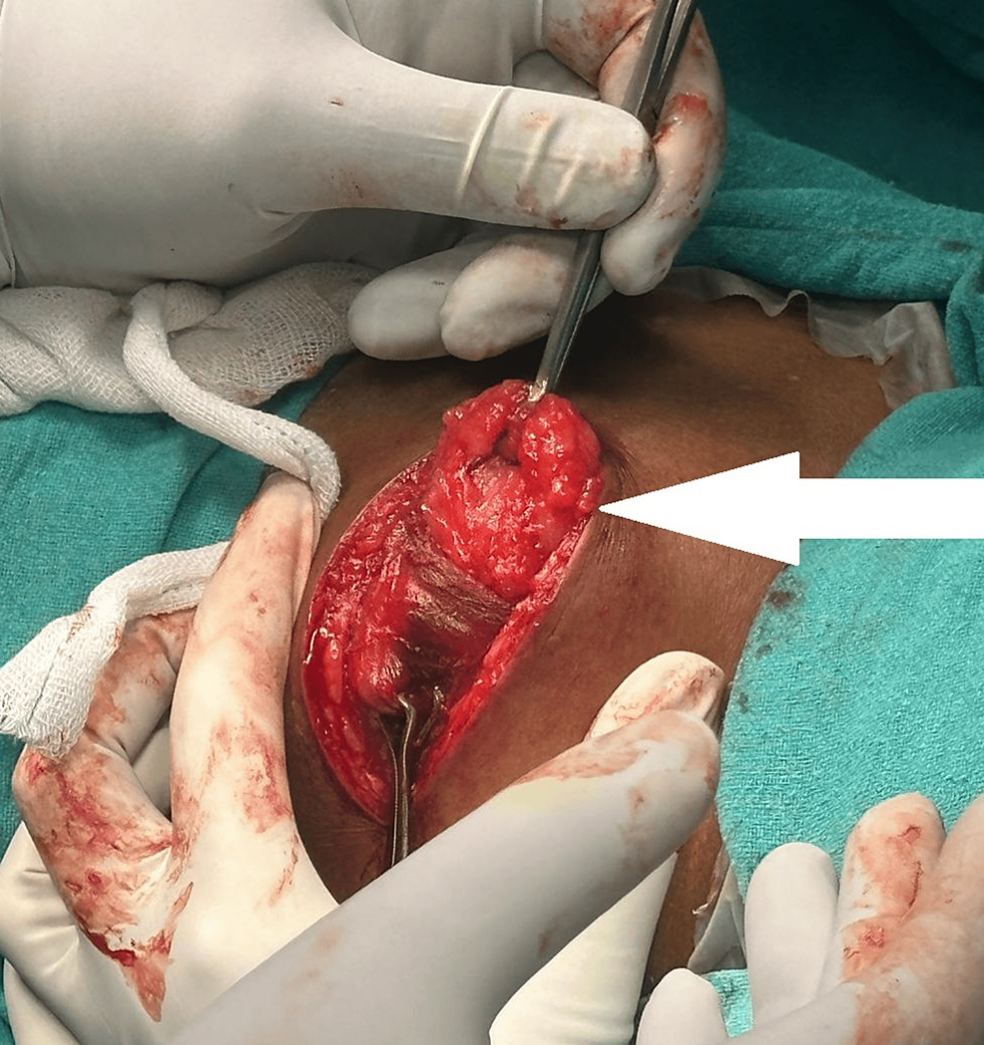
Intramuscular lipoma under surgical excision Intramuscular lipoma under surgical excision is pointed by the white arrow

**Figure 4:**
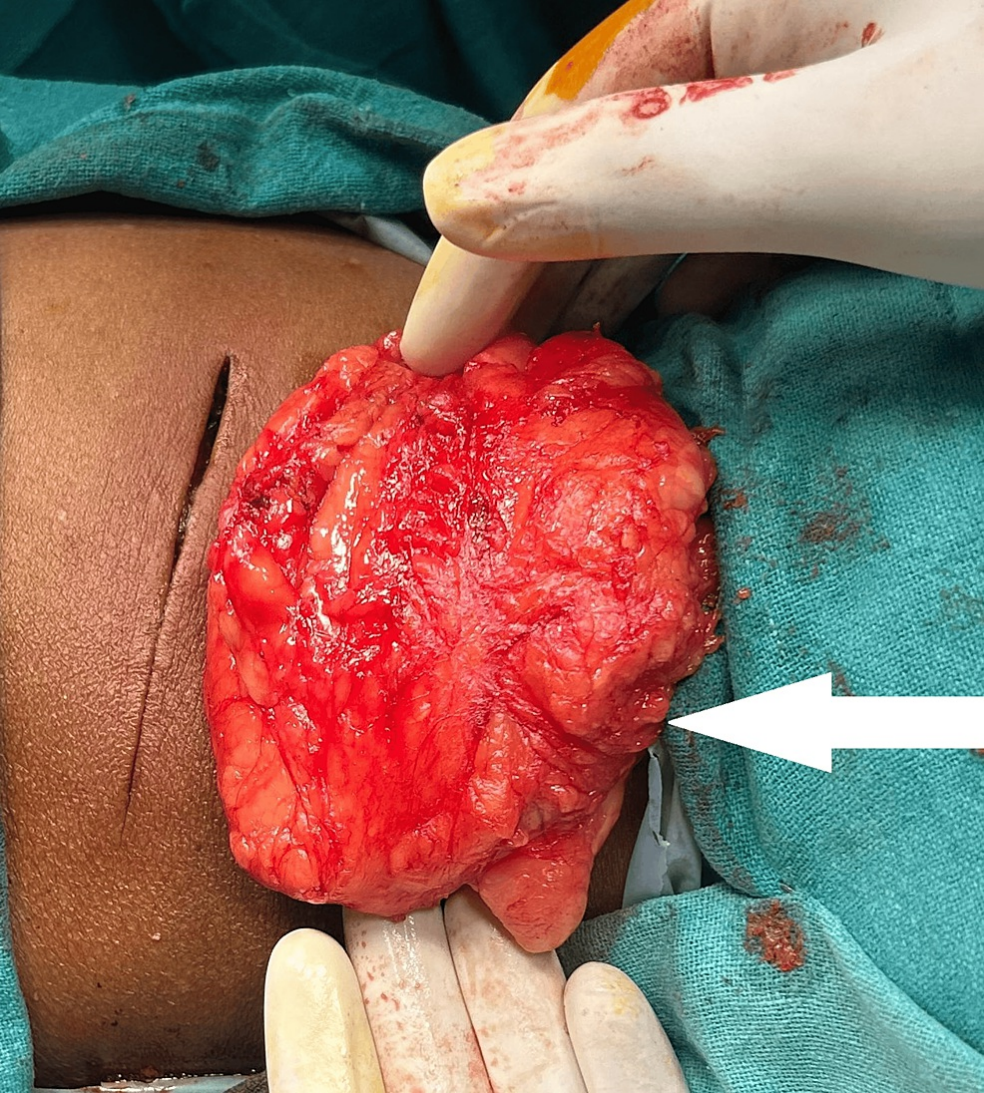
The excised specimen of lipoma The excised specimen of lipoma is pointed with the white arrow

Following the surgical excision of the intramuscular lipoma, the excised tissue specimen was sent for histopathological examination to confirm the diagnosis and ensure the complete removal of the tumor. Intramuscular lipoma under surgical excision is shown in Figure 3. The excised specimen of lipoma is shown in Figure 4.

Histopathological examination involves a pathologist's microscopic tissue sample analysis to assess its cellular composition and characteristics. In our case, the histopathological examination confirmed the presence of adipose tissue consistent with a diagnosis of lipoma, validating the MRI findings. Histopathological examination played a crucial role in confirming the nature of the lesion, ruling out any malignant potential, and ensuring complete excision. It provided valuable information for guiding further management and assessing the need for additional interventions. This case highlights the importance of considering intramuscular lipomas as a differential diagnosis for painless, slow-growing soft tissue masses. It emphasizes the role of surgical excision in symptomatic or cosmetically concerning cases. The timeline of events in the case is depicted in Table 1.

**Table 1:** The timeline of events in the case MRI: Magnetic resonance imaging

Timeline	Event
Six years ago	Initial appearance of swelling in the right lumbar region (2 cm) without pain
Present	The patient presents at AVBRH with swelling now measuring 10 x 8 x 8 cm. MRI confirms intramuscular lipoma within the right latissimus dorsi muscle.
Preoperative day	The patient admitted to AVBRH. Preoperative assessments conducted. Surgical plan discussed.
Day 1	The patient prepared for surgery under general anesthesia in the left lateral position. Surgical field was prepared and draped aseptically. A horizontal incision was made over the right lumbar region to expose swelling. A sharp dissection was performed to reach the fascia surrounding the right latissimus dorsi muscle. An encapsulated mass within the muscle was visualized. Careful dissection was made to identify and preserve nerves and blood vessels. Complete excision of the intramuscular lipoma was performed. Hemostasis maintained throughout procedure. The wound was irrigated with sterile saline solution. Closure of the wound was performed in layers using appropriate sutures. Sterile dressing was applied to surgical site. The patient was transferred to a recovery area for postoperative monitoring.
Postoperative day 1	The patient was monitored for pain and wound healing. Pain management and wound care instructions were provided.
Postoperative weeks 1-2	Scheduled follow-up appointments to assess healing progress and monitor for complications.
Recurrence monitoring (two years)	Scheduled follow-up appointments every three months for the first year, then every six months for the second year. Imaging studies (MRI or ultrasound) were conducted periodically to monitor for recurrence. The patient was instructed to report any new symptoms or changes in the surgical site between appointments.

## Discussion

Sohn et al. reported a case of intramuscular lipoma in the sternocleidomastoid muscle involving rare occurrences of intramuscular lipomas, necessitating surgical resection for complete removal [6]. In our case of a 64-year-old male patient, lipoma is in the latissimus dorsi muscle of the right lumbar region. In both instances, anatomical locations differ significantly, with the sternocleidomastoid muscle in the neck and the latissimus dorsi in the lower back. The surgical approach in the sternocleidomastoid case requires meticulous planning due to the proximity of vital structures in the head and neck region, potentially involving sacrificing attached muscles for safe removal [6]. Contrastingly, considering anatomical considerations, the surgical approach in the latissimus dorsi region may vary. Despite these variances, both cases highlight the necessity of addressing the high recurrence rate and infiltration pattern characteristic of intramuscular lipomas, emphasizing the importance of complete resection to minimize recurrence risk [3]. Both cases contribute valuable insights into managing intramuscular lipomas in rare locations, emphasizing the significance of thorough preoperative evaluation, meticulous surgical planning, and complete resection for successful outcomes [6]. Further documentation and research are warranted to enhance understanding and optimize management strategies for intramuscular lipomas across diverse anatomical sites.

A case of a 40-year-old Korean man reported by Lee et al. with an intramuscular lipoma in the chest wall is presented as a pulmonary nodule incidentally detected in the right upper lung field [7]. Our case is of a 64-year-old male with a giant intramuscular lipoma in the right lumbar region's latissimus dorsi muscle. Despite the differing locations, both cases underscore the potential asymptomatic nature of intramuscular lipomas and the importance of considering them in soft tissue mass differentials. Surgical intervention was successful in the first case via thoracoscopic excision, while our case required more extensive surgical planning due to the mass's considerable size and anatomical location [7]. Histopathological examination in both cases confirms the diagnosis of intramuscular lipomas, highlighting features such as chondroid metaplasia and mature adipocytes [7].

A case of a 71-year-old man reported by Matsumoto et al. with multiple masses around the scapular area, being a well-differentiated liposarcoma, sheds light on the association between intramuscular liposarcoma and multiple intramuscular lipomas [8]. Conversely, our case of a 64-year-old male with a singular giant intramuscular lipoma in the latissimus dorsi muscle of the right lumbar region highlights the clinical significance of giant intramuscular lipomas, given their potential for significant local mass effect and challenging surgical management. Both cases underscore the importance of meticulous clinical evaluation and histopathological examination [8]. In comparing both cases, they involve older male patients with soft tissue masses in the back region, albeit with differing characteristics.

A case of a 58-year-old man reported by Klessinger et al. mentioned a lipoma developed after decompression surgery for lumbar spinal canal stenosis [9]. Our case is of a 64-year-old male presented with a giant intramuscular lipoma in the right lumbar region without any history of spinal surgery. This contrast highlights the rarity of such tumors and underscores the importance of thorough diagnostic evaluation when encountering unusual masses in the lumbar region [9]. The recurrence of the lipoma in the 58-year-old patient is noteworthy despite macroscopically complete resection initially, the tumor recurred and required subsequent removal [9]. This recurrence suggests the aggressive nature of some intramuscular lipomas and emphasizes the challenges in achieving complete excision and preventing relapse [9].

In contrast, there is no recurrence in the two-year follow-up of our case of a 64-year-old patient, suggesting potential differences in tumor behavior or management approaches. The association between spinal surgery and the development or recurrence of intramuscular lipomas, as observed in the case of a 58-year-old man, raises important considerations regarding the etiology and pathogenesis of these tumors [9]. While such a relationship has not been previously described in the literature, it underscores the need for further research to elucidate potential causative factors and mechanisms underlying the growth and recurrence of intramuscular lipomas, particularly in the context of surgical intervention. The comparative analysis of these two cases highlights intramuscular lipomas' rarity and clinical complexity, particularly in the lumbar region. It underscores the importance of comprehensive diagnostic evaluation, careful surgical planning, and long-term follow-up in managing these challenging tumors. Furthermore, it underscores the need for further research to understand better the factors influencing their development, recurrence, and response to treatment.

## Conclusions

In conclusion, the presented case of a 64-year-old male with a giant intramuscular lipoma in the right lumbar region's latissimus dorsi muscle underscores several crucial aspects in diagnosing and managing such rare soft tissue masses. The case highlights the importance of considering intramuscular lipomas in the differential diagnosis of painless, slow-growing masses, emphasizing the significance of thorough preoperative evaluation and imaging modalities like MRI for accurate diagnosis. As demonstrated in this case, surgical intervention remains the cornerstone of treatment, necessitating meticulous dissection and complete excision to minimize recurrence risk and ensure optimal patient outcomes. Comparative analyses with other documented cases further enrich our understanding of intramuscular lipomas across diverse anatomical locations, shedding light on variations in surgical approaches, histopathological features, and potential associations with factors such as prior surgery. These insights underscore intramuscular lipomas' complexity and clinical significance, urging continued research to elucidate underlying mechanisms, optimize management strategies, and improve long-term outcomes. Ultimately, the comprehensive management of intramuscular lipomas demands a multidisciplinary approach involving close collaboration between clinicians, radiologists, pathologists, and surgeons. We can strive toward better outcomes, enhanced patient care, and further advancements in soft tissue tumor management through concerted efforts in diagnosis, treatment, and follow-up.
